# Mechanistic target of rapamycin (mTOR) regulates trophoblast folate uptake by modulating the cell surface expression of FR-α and the RFC

**DOI:** 10.1038/srep31705

**Published:** 2016-08-26

**Authors:** Fredrick J. Rosario, Theresa L. Powell, Thomas Jansson

**Affiliations:** 1 Division of Reproductive Sciences, Department of Obstetrics and Gynecology, University of Colorado Anschutz Medical Campus, Aurora, CO, USA; 2 Section of Neonatology, Department of Pediatrics, University of Colorado Anschutz Medical Campus, Aurora, CO, USA.

## Abstract

Folate deficiency in fetal life is strongly associated with structural malformations and linked to intrauterine growth restriction. In addition, limited availability of methyl donors, such as folate, during pregnancy may result in abnormal gene methylation patterns and contribute to developmental programming. The fetus is dependent on placental transfer of folate, however the molecular mechanisms regulating placental folate transport are unknown. We used cultured primary human trophoblast cells to test the hypothesis that mechanistic target of rapamycin complex 1 (mTORC1) and 2 (mTORC2) regulate folate transport by post-translational mechanisms. Silencing raptor (inhibits mTORC1) or rictor (inhibits mTORC2) markedly decreased basal folate uptake. Folate uptake stimulated by insulin + IGF-1 was mediated by mTORC2 but did not involve mTORC1. mTORC1 or mTORC2 silencing markedly decreased the plasma membrane expression of FR-α and RFC transporter isoforms without affecting global protein expression. Inhibition of the ubiquitin ligase Nedd4-2 had no effect on folate transport. In conclusion, we report for the first time that mTORC1/C2 are positive regulators of cellular folate uptake by modulating the cell surface abundance of specific transporter isoforms. We propose that regulation of placental folate transport by mTOR signaling provide a direct link between placental function, gene methylation and fetal programming.

Folate is critical for fetal development and folate deficiency is associated with fetal malformations, including neural tube defects^1^,^2^, and reduced fetal growth^3^,^4^. Consistent with these observations, folate supplementation in women with folate deficiency decreases the incidence birth defects and increases birth weight^2^,^5^. Gene methylation and other forms of epigenetic regulation of key metabolic pathways at critical windows of intrauterine development have been implicated as a mechanism underlying developmental programming of metabolic and cardiovascular disease^6^. Limited availability of methyl donors, such as folate, may result in abnormal gene methylation patterns and contribute to developmental programming^7^. In addition to maternal folate intake, fetal folate availability is critically dependent on the capacity of the placenta to transport folate and it is possible that reduced placental folate transport contributes to decreased fetal folate availability. However, the molecular mechanisms regulating placental folate transport are largely unknown.

Folates are small (Mr~500), hydrophilic, anionic molecules that are transferred across the plasma membrane mediated by specific transport mechanisms, including the Folate Receptor-α (FR-α), Proton Coupled Folate Transporter (PCFT), and Reduced Folate Carrier (RFC)^8^. FR-α is anchored to the plasma membrane by glycophosphotidylinositol (GPI) with a molecular mass of ~28 to 40 kDa; it has a greater affinity for oxidized folate (folic acid) than reduced forms (methyltetrahydrofolate)^8^. FR-α transports folate via receptor-mediated endocytosis/exocytosis and functions at a neutral to mildly acidic pH. FR-α moves between the cell surface^9^ and endocytic compartments via a clathrin-independent and Cdc42-dependent pinocytic pathway^10^. PCFT *(SLC46A1)* has a molecular weight of 50 to 65 kDa, depending upon the extent of glycosylation^11^. PCFT mediates the co-transport of folate and protons, and has optimal activity at low pH with similar affinity for oxidized and reduced forms of folate^12^. RFC is an anionic exchanger, mediating the cellular uptake of folate in exchange for anions such as organic phosphates. RFC has been proposed to be the major route of delivery of folate to systemic tissues at physiologic pH. FR-α, PCFT, RFC have all been shown to be expressed and active in the human placenta^9^,^13^,^14^,^15^. These transporters are believed to act in coordination to ensure the vectorial transfer of folate from maternal to fetal circulation^9^.

Our understanding of the mechanisms regulating folate transporters is limited. Mono methyl fumarate, a compound used to treat psoriasis, inhibits PCFT mediated folate transport in retinal Muller cells^16^. Folate malabsorption across the basolateral plasma membrane of the colon epithelium^17^ and pancreatic acinar cells^18^ in association to chronic ethanol ingestion is mediated by down regulation of RFC and PCFT transporters. Acute folate over supplementation results in a significant decrease in intestinal and renal folate uptake due to down-regulation of the expression of FR, RFC and PCFT, mediated by post-transcriptional or translational mechanisms^19^,^20^. Nuclear respiratory factor (NRF1) binding protein functions as a major inducible transcriptional regulator of PCFT gene expression in the intestine^21^. Insulin has been reported to increase folate uptake in cultured skin fibroblast cells isolated from fetal rats^22^. In addition, Vitamin D (3) binds to a Vitamin D response element in the PCFT gene, resulting in increased PCFT expression, and enhanced cellular folate uptake in the intestine^23^.

The mTOR signaling pathway regulates gene transcription and protein translation in response to nutrient and growth factor availability, resulting in changes in cell metabolism and growth^24^,^25^. mTOR exists in two complexes, mTOR Complex 1 (mTORC1) and 2 (mTORC2). One of the key differences between these two complexes is that mTOR associates to the protein raptor in mTORC1 and to rictor in mTORC2^26^. DEPTOR, a protein containing two DEP (Dishevelled, Egl-10, Pleckstrin) domains, is an endogenous inhibitor of both mTORC1 and 2 signaling^27^. We have demonstrated previously that mTOR signaling is a positive regulator of system A and System L amino acid transporters in cultured human primary trophoblast cells^28^,^29^. However, regulation of folate transporters by mTOR has not been reported previously, in any cell type. Here, we tested the hypothesis that mTORC1 and mTORC2 regulate folate transport by post-translational mechanisms. To this effect, using siRNA approaches we silenced raptor (to inhibit mTORC1 function), rictor (inhibits mTORC2 function), raptor + rictor (inhibits mTORC1 and mTORC2) or DEPTOR (to activate both mTORC1 and mTORC2) in cultured primary human trophoblast cells and subsequently determined folate uptake and protein expression of FR-α, PCFT and RFC.

## Materials and Methods

Term placental tissue was collected from healthy pregnant women (n = 6, Table 1) at delivery after written informed consent. All study subjects were taking prenatal vitamins containing folic acid during pregnancy. The study was approved and carried out in accordance with the approved guidelines by the Institutional Review Board of University of Colorado. Samples and medical information were added to a tissue repository approved by the Colorado Multiple Institutional Review Board (14-1073) and de-identified samples and clinical information were provided for use in this study. Cytotrophoblast cells were isolated from normal term placentas and cultured *in vitro*^30^. Briefly, placental tissue was minced and then subjected to trypsin (0.25%, Invitrogen, Carlsbad, CA) and DNase (0.2 mg/ml, Sigma-Aldrich, St. Louis, MO) digestion. The cytotrophoblast cells were then isolated by a discontinuous Percoll gradient and diluted to 3 × 10^6^ cells/ml in cell culture media made up of a 1:1 mixture of Dulbecco’s modified Eagle’s medium (DMEM) containing 25 mM glucose and Ham’s F-12 Nutrient Mixture containing 10 mM glucose plus 10% fetal bovine serum (FBS), 50 μg/ml gentamicin, 60 μg/ml benzylpenicillin, and 100 μg/ml streptomycin. The cells were then further diluted and plated in either 60 mM culture dishes (~7.5 × 10^6^ cells/dish for Western blot analysis) or 6-well plates (for ^3^H-5-Methyltetrahydrofolic acid (MTHF) uptake experiments; ~2 × 10^6^ cells/well for rapamycin treatment or ~2.75 × 10^6^ cells/well for RNAi mediated gene silencing) and cultured in 5% CO_2_, 95% atmosphere air at 37 °C for 90 h.

### RNA Interference-mediated silencing

Dharmafect 2 transfection reagent (Thermo Scientific, Rockford, IL) and small interfering RNAs (siRNAs) (Sigma-Aldrich, St. Louis, MO), targeting raptor (100 nM; sense, 5′CAGUUCACCGCCAUCUACA), rictor (100 nM; sense, 5′CGAUCAUGGGCAGGUAUUA), DEPTOR (SASI_1297010-H/5582, 1297011-H) or Nedd4-2 (100 nM; 5′CCCUAUACAUUUAAGGACU) were used. Control cells were transfected with a non-coding scrambled sequence (100 nM; sense: 5′GAUCAUACGUGCGAUCAGATT). siRNAs were added to cultured PHT cells (~3.75 × 10^6^ cells/well in 6 well plate; ~7.5 × 10^6^ cells in 60 mm dish) after 18 h in culture, incubated for 24 h^31^ and subsequently removed and replaced by fresh medium. At 90 hours in culture, efficiency of target silencing was determined at the protein and functional levels using Western blot.

### Growth factor treatment

The current study was not designed to specifically explore the possibility that insulin and IGF-1 may have distinct effects on folate uptake. Instead our approach was to achieve a maximal growth factor response using insulin and IGF-1 together. PHT cells were incubated in 5.8 ng ml^−1^ insulin (Sigma-Aldrich) and 300 ng ml^−1^ insulin-like growth factor 1 (IGF-1; Sigma-Aldrich) from 42 to 90 h in culture. The insulin concentration used in these experiments corresponds to normal postprandial insulin levels in pregnant women^32^. The IGF-1 concentration used constitutes physiological concentrations of IGF-1 in third trimester maternal serum^33^.

### MTHF uptake assay

[3′, 5′, 7, 9-^3^H]-Methyltetrahydrofolic acid uptake in PHT cells was determined at 90 hours in culture. In brief, transport experiments were performed in a buffer with the following composition (in mM): 150 NaCl, 10 HEPES (*N*-2-hydroxyethylpiperazine-*N*′-2-ethanesulfonic acid) and 5.6 d (+) glucose (pH 7.4). Initially, the culture medium was removed, cells were washed with buffer at 37 °C and uptake was initiated by the addition of 1 ml of buffer at 37 °C containing 30 nM [^3^H] MTHF (Moravek Biochemicals, Brea, CA). Uptakes were stopped after 80 min by removing the incubation medium followed by washing the cells twice with 1 ml of ice-cold phosphate buffer saline (PBS, pH 7.4). Non-mediated uptake was determined in the presence of 1.5 mM unlabelled MTHF^15^. Cell protein concentration was assessed by BCA protein assay kit (Bio-Rad, CA) and uptakes expressed as pmol MTHF/(mg protein × time).

### Isolation of MVM from trophoblast cells

Microvillous plasma membranes (MVM) were isolated from total cell lysates of cultured PHT cells using differential centrifugation and Mg^2+^ precipitation as described previously^29^. In brief, cells were lysed, scraped off the plate, homogenized and centrifuged. The pelleted crude membrane fraction was resuspended and 12 mM MgCl_2_ was added. The mixture was stirred slowly for 20 min on ice and then centrifuged. The supernatant containing MVM was centrifuged at 125,000 g for 30 min and the final pellet was resuspended. Protein concentration was determined using the Bradford assay. Protein expression of the MVM marker alkaline phosphatase was determined in MVM vesicles and cell lysate by Western blot. The MVM enrichment, as determined by the ratio of alkaline phosphatase expression in MVM over total cell lysates, was similar in MVM isolated from control (9.7 ± 1.4 fold), raptor (8.0 ± 1.5 fold) and rictor silenced cells (11.3 ± 2.9 fold).

### Western blotting

For immunoblotting, cells were lysed in buffer containing phosphatase and protease inhibitors. Subsequently, cells were scraped, collected, and sonicated. Proteins in cell lysates and MVM were separated by electrophoresis. Western blotting was carried out as described^28^. Protein expression of FR-α, RFC and PCFT was determined in total cell lysates and MVM preparations using commercial antibodies (Santa Cruz Biotechnology, TX). Anti-β actin was from Sigma-Aldrich, St. Louis, MO. Target expression was corrected for any differences in protein loading by using beta-actin expression. For each protein target the mean density of the control sample bands was assigned an arbitrary value of 1. All individual densitometry values were expressed relative to this mean.

### Immunofluorescence

PHT were cultured on chamber slides (Lab-Teck) for 90 h. At 18 h of culture cells were transfected with scramble siRNA or raptor siRNA. At 90 h cells were fixed with 4% paraformaldehyde at room temperature for 30 min and were blocked using 5% new born calf (NCS) serum in PBS for 1 hour followed by anti-RFC (generated in goat) for 1 hour. After several washes, cells were incubated in an Alexa Fluor 568-conjugated rabbit anti-goat IgG for 1 h at room temperature. After three washes in PBS, coverslips were mounted in a drop of Vectashield (Vector Laboratories, Burlingame, CA, USA). Confocal microscopy was performed using Zeiss LSM 780 microscope at 63x magnification using oil immersion. Images were captured in the same laser settings with four Z-step of 0.4 um.

### Data presentation and statistics

The number of experiments (n) represents the number of placentas studied. Data are presented as means ± S.E.M. or +S.E.M. Statistical significance of differences was assessed using paired Student’s t-test (comparisons between two groups) or repeated-measures (RM) ANOVA with Tukey’s post-hoc test (comparisons between three groups). The rationale for this approach is that the different treatment groups are related and not independent because PHT cells isolated from the same placenta subjected to two or three different treatments were compared. A P value < 0.05 was considered significant.

## Results

### mTORC1 and mTORC2 silencing inhibits folate uptake in PHT cells

**Figure 1:**
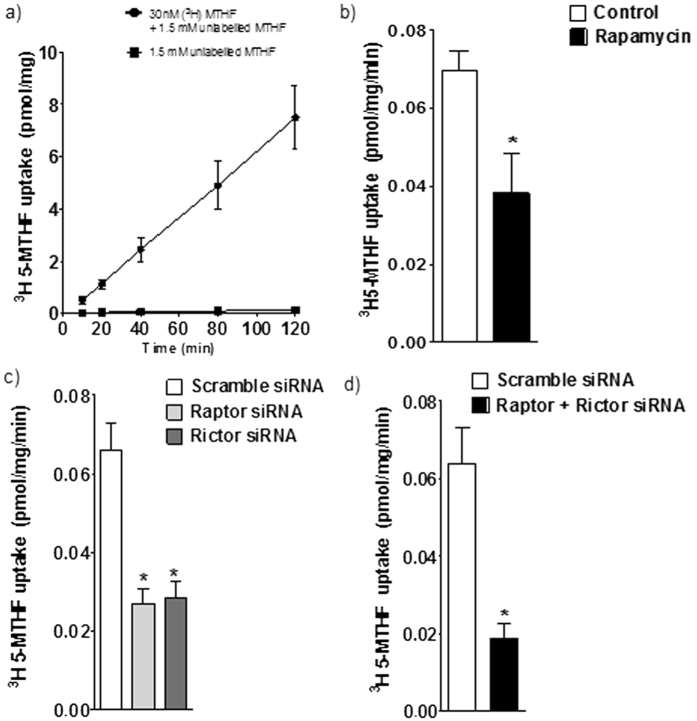
Regulation of PHT folate uptake byTORC1 and 2. **(a)** Time course of ^3^H-5-MTHF uptake in the absence and presence of 1.5 mM unlabeled MTHF in cultured human primary trophoblast (PHT) cells. Uptakes were linear for at least 120 min. Values are given as means ± SEM. n = 4. **(b)** Inhibition of mTOR by 100 nM rapamycin decreased the basal folate uptake in PHT cells. **(c)** Effect of silencing raptor (mTORC1 inhibition) or rictor (mTORC2 inhibition) on folate uptake. (**d**) Effect of simultaneous silencing of raptor and rictor on basal folate uptake. Uptake of [3′, 5′, 7, 9-^3^H]-Methyltetrahydrofolic acid was measured for 80 minutes at 37 °C. Values are means + SEM; n = 3–6. *P < 0.05 versus control; paired Student’s t test (**b,d**) RMANOVA with Tukey–Kramer multiple comparisons post hoc test; means without a common letter differ significantly (P < 0.05).

The uptake of methyltetrahydrofolate (MTHF) in PHT cells was linear for at least 120 minutes (Fig. 1a) at pH 7.4, and subsequent transport studies were performed at 80 minutes. In order to study the effect of mTOR inhibition on trophoblast folate uptake, after 66 h in culture, trophoblast cells were incubated with 100 nM rapamycin or vehicle (0.02% DMSO) for 24 h, and the uptake of [^3^H]-5 MTHF was then measured. As shown in Fig. 1b, rapamycin reduced MTHF uptake by 45% (n = 4; P < 0.05) compared to control. Because some observations suggest that rapamycin may also inhibit mTORC2^34^, we proceeded by using siRNA mediated silencing of raptor and rictor to specifically inhibit mTORC1 and 2, respectively. We have previously reported that this approach results in 60–70% decrease in raptor/rictor protein expression and a marked functional inhibition of mTORC1 and 2 signaling pathways without affecting syncytialization, viability or apoptosis^29^. Silencing of raptor (−57%, n = 6; P < 0.001) or rictor (−54%, n = 6; P < 0.001) inhibited basal levels of [^3^H]-5 MTHF uptake (Fig. 1c) as compared to PHT’s transfected with scramble siRNA. Silencing of both raptor and rictor resulted in an even more pronounced decrease in folate uptake (Fig. 1d, −83%, n = 4; P < 0.001) compared to scramble siRNA.

### DEPTOR silencing increases folate uptake in PHT cells

**Figure 2:**
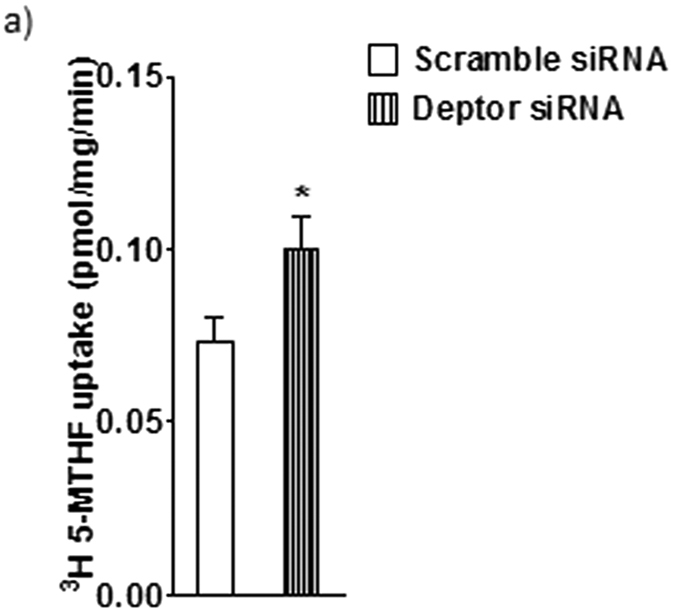
Activation of mTORC1 and 2 stimulates folate uptake in PHT cells. mTORC1 and 2 were activated by silencing of DEPTOR and folate transport was studied by uptake of ^3^H-5-MTHF uptake in PHT cells. Values are means + SEM; n = 6. *P < 0.05 versus control; paired Student’s t test.

To further establish mTOR regulation of PHT folate transport we tested the hypothesis that activation of mTOR stimulates folate uptake. DEPTOR is a constituent of both mTOR complexes and is considered a negative regulator of mTOR function^27^. Thus, to activate mTORC1 and mTORC2 signaling, we silenced DEPTOR in PHT cells. This approach results in 70% decrease in target protein expression and a marked functional activation of mTORC1 and 2 signaling pathways without affecting syncytialization, viability or apoptosis^35^. DEPTOR silencing increased MTHF uptake (+36%, P < 0.04; n = 6, Fig. 2a) compared to control. This data confirms that mTOR signaling is a positive regulator of trophoblast folate uptake.

### Growth factors stimulate folate uptake mediated by mTORC2 signaling

**Figure 3:**
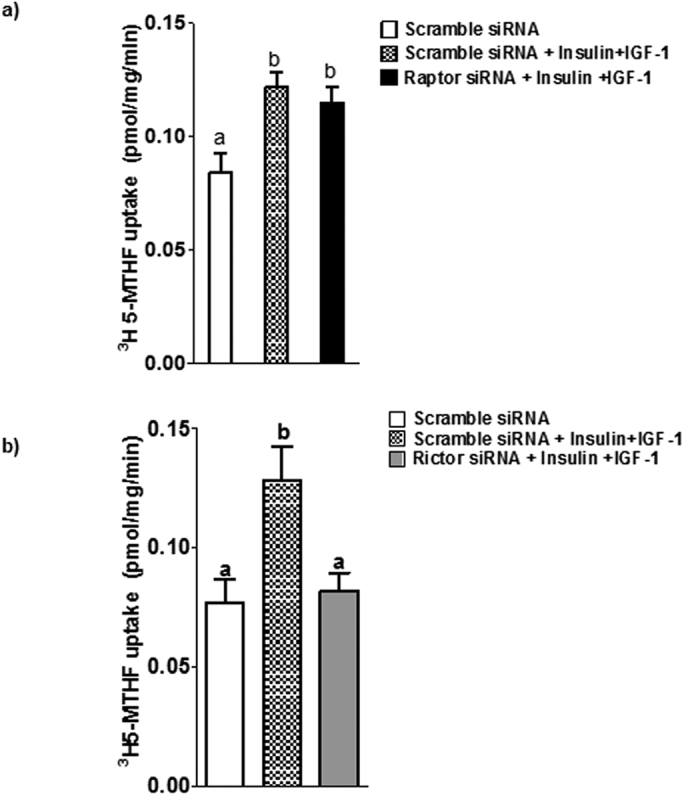
Growth factors stimulates folate uptake in PHT cells mediated by mTORC2. (**a**) The stimulatory effect of growth factors [Insulin (5.8 ng ml^−1^) + IGF-1 (300 ng ml^−1^)] on ^3^H-5-MTHF uptake was not significantly influenced by raptor silencing. **(b)** In contrast, rictor silencing in PHT cells prevented growth factor-stimulated folate uptake. Values are means + SEM; n = 5. Means without a common letter differ significantly (P < 0.05) by RMANOVA with Tukey–Kramer multiple comparisons post hoc test.

We next studied the role of mTOR signaling in mediating the effect of growth factors on folate uptake. Trophoblast cells were incubated with insulin and IGF-1 for 48 h (42–90 h) and folate uptake was measured at 90 h. As shown in Fig. 3a, insulin and IGF-1 stimulated [^3^H]-5 MTHF uptake (+% 44, *P* < 0.01; *n* = 5). Growth factor stimulated folate uptake was unaffected by raptor silencing (mTORC1 inhibition, Fig. 3a). However, rictor silencing completely prevented stimulation of folate uptake by growth factors (Fig. 3b, *P* < 0.05; n = 5), suggesting that regulation of folate uptake by growth factors is dependent on mTORC2 signaling in human primary trophoblast cells.

### mTORC1 and mTORC2 do not regulate global expression of FR-α, PCFT, and RFC

**Figure 4:**
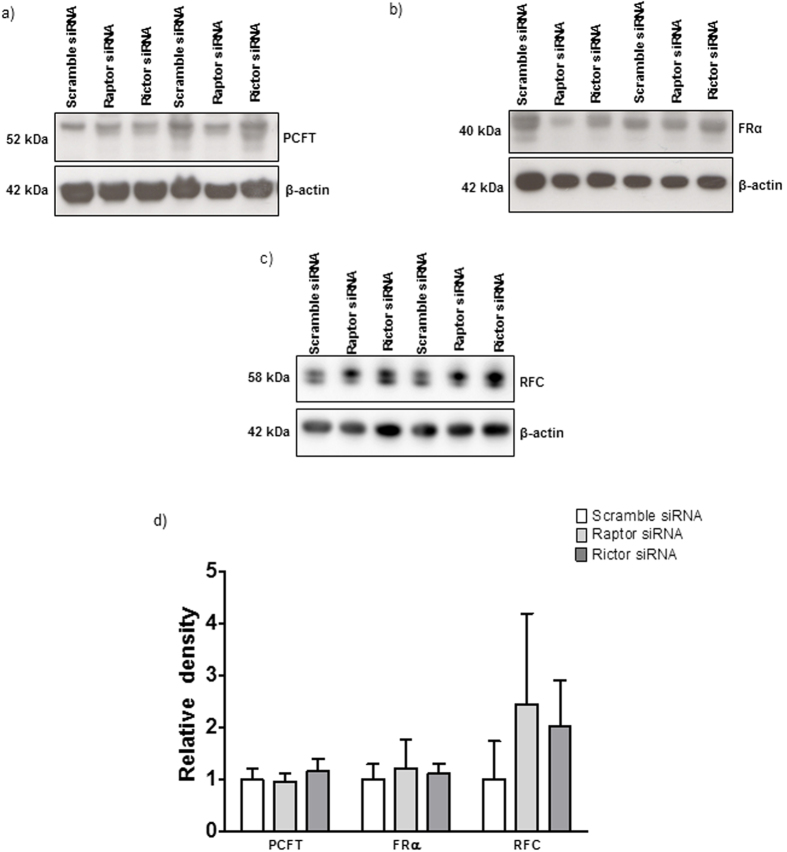
mTOR inhibition does not alter the global protein expression of folate transporter isoforms in total PHT cell lysates. Representative western blots are shown for (**a**) PCFT (**b**) FR-α and (**c**) RFC in cell lysates of PHT cells transfected with scramble, raptor or rictor siRNA. Equal loading was performed. The histogram (**d**) summarizes the western blot data. Values are means + SEM, n = 3.

Cellular folate uptake is mediated by specific transport mechanisms, in particular FR-α, PCFT, and RFC^36^. Because mTORC1 signaling is well established to regulate mRNA translation^37^,^38^, we first explored effects of mTOR inhibition on folate transporter isoform expression in cell lysates. As shown in Fig. 4, protein expression of FR-α, PCFT, and RFC in total cell lysates was unaffected by raptor or rictor silencing. These data indicate that mTOR signaling regulates trophoblast folate uptake by post-translational mechanisms.

### mTORC1 and mTORC2 signaling regulate trophoblast folate transporter trafficking

**Figure 5:**
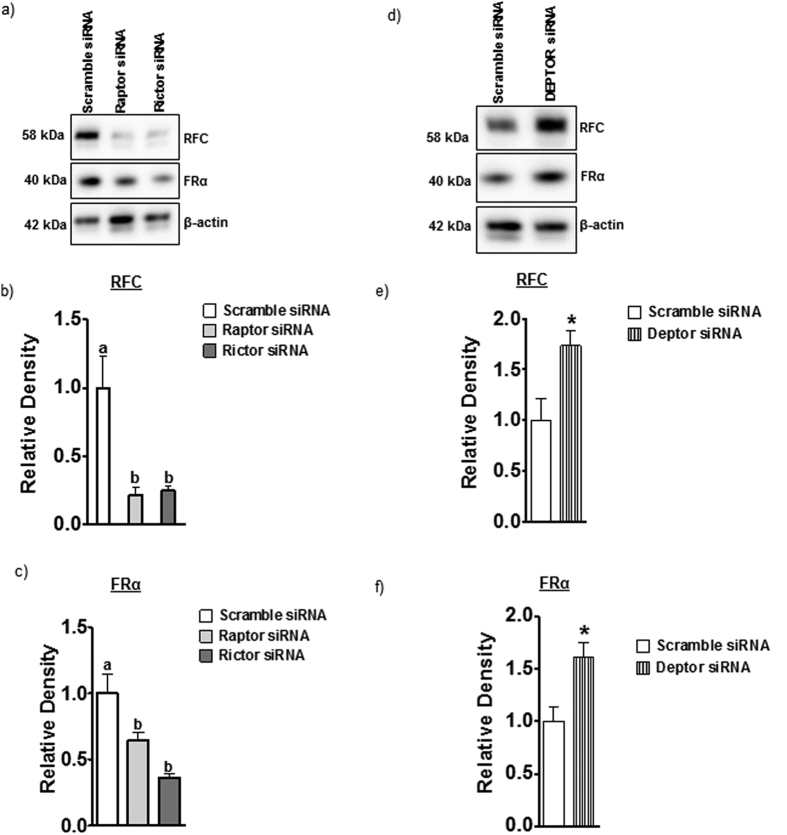
mTORC 1 and mTORC2 regulate the protein expression of RFC and FR-α in MVMs isolated from PHTs. Representative western blots are shown for RFC and FR-α in cell lysates and MVMs of control and raptor, rictor (**a**) or Deptor silenced PHT cells (**d**). Equal loading was performed. The histograms (**b,c,e,f**) summarize the data. Expression in control cells for each isoform was arbitrarily assigned a value of one. Values are means + SEM; n = 3, *P < 0.05 versus control; paired Student’s t test.

**Figure 6:**
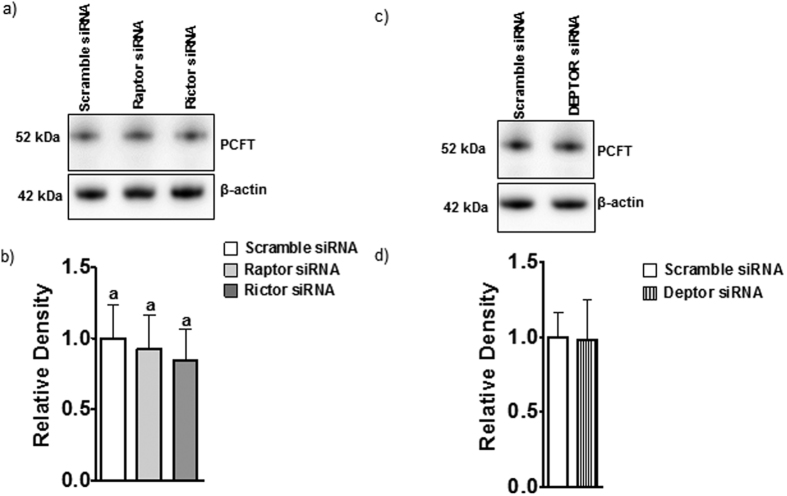
Protein expression of PCFT in MVMs isolated from PHTs is not regulated by mTORC1 or 2. Representative western blots (**a,c**). Equal loading was performed. The histograms (**b,d**) summarize the data. Expression in control cells for each isoform was arbitrarily assigned a value of one. Values are means + SEM; n = 3, *P < 0.05 versus control; paired Student’s t test.

To study changes in cell surface expression we isolated a microvillous plasma membrane (MVM) fraction from PHT cells and determined MVM protein expression of folate transporters in response to raptor or rictor silencing. Raptor or rictor silencing decreased the MVM expression of FR-α and RFC (Fig. 5a–c) without significantly affecting MVM PCFT expression (Fig. 6a,b). Activation of mTOR signaling by DEPTOR silencing increased the protein expression of FR-α and RFC in MVM (Fig. 5d–f) but did not affect the expression of PCFT in MVM (Fig. 6c,d). Collectively, these results demonstrate that mTORC1 and mTORC2 regulate cellular folate uptake by modulating the cell surface expression of FR-α and RFC, which is reminiscent of the mechanism by which mTOR regulates amino acid transport in PHT cells^29^.

### Cellular localization of RFC protein expression in PHT cells with mTORC1 inhibition

**Figure 7:**
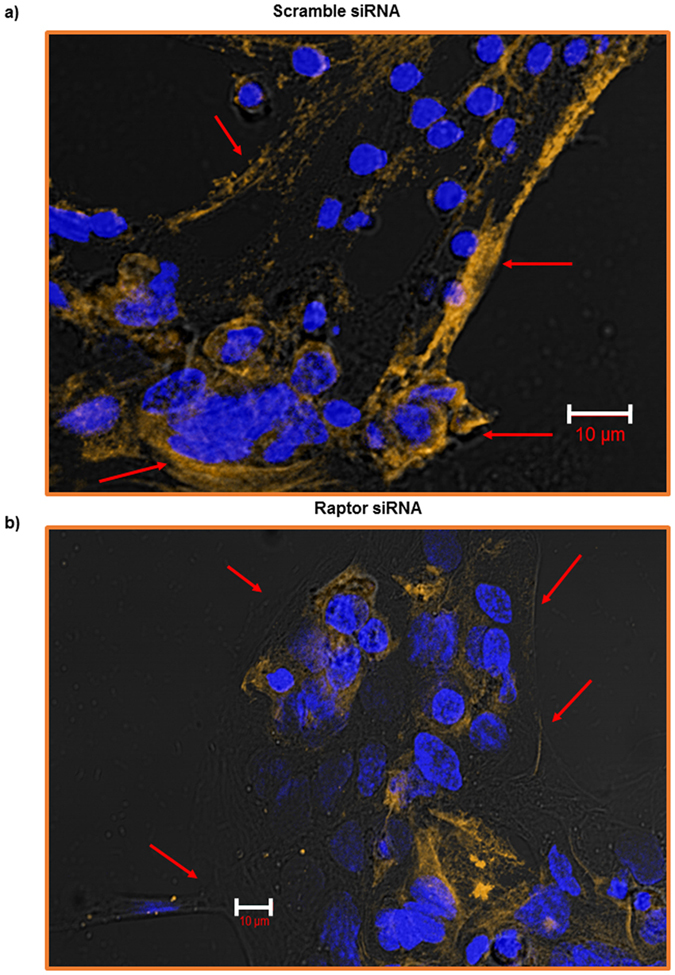
Cellular localization of RFC protein expression in PHT cells with mTORC1 inhibition. Trophoblast cells were transfected at 18 h in culture with scramble (**a**) or raptor (**b**) siRNA. At 90 h in culture, cells were fixed and RFC expression (yellow) was visualized using immunofluorescence. The data are from a representative experiment, and similar results were obtained from two other experiments. Nuclei were counterstained using DAPI (4′,6-diamidino-2-phenylindole) (blue). Scale bars represent 10 μm.

To further confirm that inhibition of mTORC1 influences membrane trafficking of RFC, we used confocal microscopy to determine the cellular localization of RFC protein in cultured PHT cells. We observed that in response to raptor (Fig. 7) silencing RFC remained in the cytosol and was not trafficked to the PHT plasma membrane.

### Nedd4-2 silencing did not influence folate uptake

**Figure 8:**
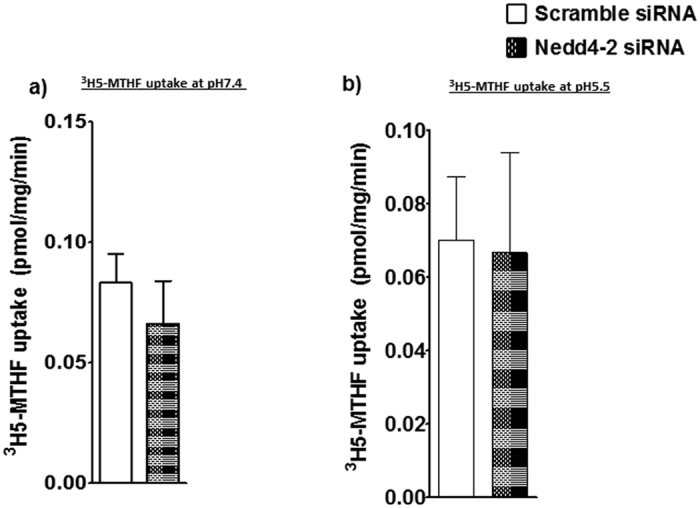
Nedd4-2 silencing does not influence PHT folate uptake. ^3^H-5-MTHF uptake in PHT cells was unaffected by Nedd4-2 silencing at pH 7.4 (**a**) and pH 5.5 (**b**). Values are means + SEM; n = 5, paired Student’s t test.

Ubiquitination is a post-translational modification that has been shown to be involved in regulating the plasma membrane expression of mammalian transporters, in particular ENaC^39^. Nedd4-2 is one of the E3 ubiquitin ligases that catalyzes ubiquitination of plasma membrane transporters, thereby controlling their cell surface expression^39^. Membrane trafficking of specific system A (SNAT 2) and system L (LAT1) amino acid transporter isoforms in primary human trophoblast cells is regulated by Nedd4-2 dependent ubiquitination^35^. Here we tested the hypothesis that Nedd4-2 mediated ubiquitination is required for mTOR regulation of folate transporter trafficking by silencing Nedd4-2 and measuring folate uptake in PHT cells. As shown in Fig. 8, Nedd4-2 silencing did not alter folate uptake as compared to control, suggesting that Nedd4-2 is not involved in the regulating trafficking of folate transporter in PHT cells.

## Discussion

We utilized gene-silencing approaches to acquire functional data in human primary trophoblast cells, which has allowed us to obtain specific mechanistic information of physiological relevance about the role of mTORC1 and mTORC2 signaling pathways in regulating placental folate transport. We report for the first time, in any cell type, that both mTORC1 and mTORC2 signaling positively regulates cellular folate uptake in cultured primary human trophoblast cells. We speculate that regulation of placental folate transport by mTOR may alter the availability of methyl donors in the fetus, thereby providing a direct link between placental function, gene methylation and fetal programming.

The mTORC1 signaling pathway influences cell function and growth by controlling translation initiation, the rate limiting step of protein synthesis^40^. Furthermore, mTORC1 also regulates the transcription of a large number of genes^25^,^41^,^42^. Our data show that mTORC1 and mTORC2 regulate the cellular folate uptake in primary human trophoblast cells without affecting global protein expression of transporter isoforms in total cell lysates, implicating a post-translational mechanism. Using plasma membranes isolated from cultured primary trophoblast cells subsequent to raptor or rictor silencing, we show that mTORC1 or mTORC2 inhibition markedly decrease the abundance of folate transporter isoforms at the cell surface indicating that mTOR modulates transporter trafficking to and/or from the plasma membrane. The effect of inhibition of the mTOR signaling pathways was transporter specific, involving RFC and FRα, but not PCFT. Importantly, activation of mTORC1 and mTORC2 by DEPTOR silencing increased the plasma membrane expression of FRα and RFC, confirming a role of mTOR signaling in regulating plasma membrane folate transporter abundance. Thus, both mTORC1 and mTORC2 signaling pathways constitute powerful positive regulators of trophoblast cell folate uptake, which is mediated by modulation of cell surface abundance of a specific subset of transporter isoforms.

We have previously reported that mTORC1 and mTORC2 regulate human trophoblast amino acid transporters by modulating the cell surface abundance of specific transporter isoforms^29^. Furthermore, ubiquitination regulates degradation of cellular proteins by the ubiquitin–proteasome protein degradation pathway, controlling protein half-life and expression levels. This process involves sequential action of three distinct enzymes. NEDD4-2 is an ubiquitin ligase shown to be involved in the degradation of SNAT-2 in adipocytes^43^ and we have previously reported a mechanistic link between mTOR inhibition, activation of NEDD4-2, increased SNAT-2 ubiquitination and decreased system A amino acid transport activity in cultured primary human trophoblast cells^35^. However, Nedd4-2 is not involved in the regulation of folate transport. The exact molecular mechanism involving mTOR regulation of folate transport remains to be established.

Because fetal development and growth are critically dependent on folate availability, regulation of placental folate transport by mTORC1 and 2 signaling may constitute an important molecular link between maternal nutrition and fetal growth. Placental mTOR signaling has been reported to be inhibited in human intrauterine growth restriction^44^,^45^,^46^ and in animal models of maternal undernutrition^47^,^48^. The findings in the present study therefore raise the possibility that placental mTOR mediated dysregulation of placental folate transport plays a role in the development of abnormal fetal growth. Folate deficiency is also associated with fetal structural malformations, including neural tube defects^49^. Although maternal folate supplementation has decreased the incidence of these malformations^50^, a large number of these structural birth defects occur every year. Thus, optimizing maternal folate level may not be sufficient to prevent all folate dependent structural malformations and it may be speculated that mTOR dependent down-regulation of placental folate transport early in pregnancy may be involved in the development of these birth defects.

It is well established that IUGR is associated with an increased risk of a variety of diseases, including obesity, diabetes and cardiovascular disease, later in life^6^,^51^. Although the exact molecular mechanisms underlying fetal programming of adult disease remain to be established, a body of evidence suggests that epigenetic mechanisms are involved^52^,^53^. It is possible that inhibition of placental mTOR signaling in IUGR^44^,^46^,^47^,^48^ results in decreased placental folate transport, contributing to limited fetal folate availability in this pregnancy complication. Given that folates are obligatory cofactors for the provision of methyl groups for epigenetic regulation of gene expression, this would potentially constitute one mechanism underlying altered gene methylation in IUGR, linking placental function to fetal programming.

To the best of our knowledge, this is the first report, in any cell, to demonstrate that mTOR signaling regulates cellular folate uptake. It is possible that this regulatory mechanism is present in other cell types as well and our findings may therefore have broad biological significance. mTOR signaling is well established to promote cell metabolism, growth and proliferation in response to increased nutrient availability and activation of growth factor signaling by increasing protein translation and the expression of a subset of genes. We have previously shown that mTOR is a positive regulator of cellular amino acid uptake^28^,^29^, thereby directly linking the rate of protein synthesis to amino acid availability. Similarly, our observations in the current study suggest that mTOR signaling directly links increased cellular metabolism, growth and proliferation to enhanced cellular uptake of folate, which is required for methylation reactions, DNA repair and *de novo* DNA synthesis.

In conclusion, we demonstrate that mTORC1 and mTORC2 signaling regulates uptake in primary human trophoblast cells by altering the cell surface expression of folate receptor-α and the reduced folate carrier. This is the first report of mTOR modulating of folate transport in any cell type and may have broad implications for the regulation of folate uptake in other human cells. Because placental mTOR activity is decreased in human intrauterine growth restriction, our data are consistent with the possibility that placental mTOR inhibition decreases the availability of methyl donors in the fetus, thereby providing a direct link between placental function, gene methylation and fetal programming.

## Additional Information

**How to cite this article**: Rosario, F. J. *et al*. Mechanistic target of rapamycin (mTOR) regulates trophoblast folate uptake by modulating the cell surface expression of FR-α and the RFC. *Sci. Rep*. **6**, 31705; doi: 10.1038/srep31705 (2016).

## Figures and Tables

**Table 1:** Clinical characteristics of study subjects.

Subject	Maternal age (years)	Gestational age (weeks)	BMI (kg/m^2^)	Placental weight (gm)	Birth weight (gm)	Maternal blood pressure (mm/Hg)		Fetal sex	Mode of delivery
						Systolic	Diastolic		
1	38	38.43	24.30	724	3019	124	84	M	C-section
2	28	39.57	24.93	750	3081	126	84	F	C-section
3	29	39.14	19.40	801	3163	115	60	F	C-section
4	30	39.86	20.00	700	3088	100	68	M	C-section
5	30	38.57	21.80	863	3073	108	76	M	C-section
6	34	39.00	22.45	747	3029	112	70	M	C-section
Mean ± S.E.M.	31.5 ± 1.5	39.09 ± 0.23	22.1 ± 0.9	764 ± 24	3075 ± 21	114 ± 4	74 ± 4		
